# Rapid and Effective Way to Synthesize Highly Crystalline Nanosized SAPO-34 Particles

**DOI:** 10.3390/nano12224086

**Published:** 2022-11-20

**Authors:** Irina Shamanaeva, Svetlana Strelova, Marina Solovyeva, Aleksandra Grekova

**Affiliations:** Boreskov Institute of Catalysis SB RAS, Lavrentiev Ave., 5, 630090 Novosibirsk, Russia

**Keywords:** SAPO-34, dry gel conversion, steam-assisted crystallization, nanocrystals, coated adsorber

## Abstract

SAPO-34 nanocrystals with sizes of 50–150 nm were obtained via steam-assisted crystallization (SAC) for 5 h at 200 °C from two types of aluminum precursors—aluminum isopropoxide and boehmite. A reaction mixture composition with a small amount of organic template tetraehylammonium hydroxide (TEAOH) was used with the molar ratio TEAOH/Al_2_O_3_ = 1/1. The alumina precursor type and duration of the SAC (5 and 24 h) on the crystal size, texture, and acid properties were investigated. The SAPO-34 nanocrystals that we obtained possess a large micropore volume of 0.22–0.24 cm^3^/g and a specific surface area of 651–695 m^2^/g. When the crystallization was prolonged for up to 24 h, a SAPO-18 structure appeared, but the micropore and mesopore volumes changed insignificantly. Using boehmite as the aluminum precursor led to higher mesoporosity of the material but a little bit lower acidity when compared with the samples prepared from aluminum isopropoxide. In addition, the method proposed was used for preparing a SAPO-34-coated aluminum adsorber heat exchanger. Thus, the synthesis method proposed is affordable and effective to prepare SAPO-34 highly crystalline nanoparticles, with no need for post-synthetic procedures as the mother liquor separation from nanocrystals.

## 1. Introduction

Silicoaluminophosphates (SAPOs) are vital members of the molecular sieve family. These zeolite-like materials consist of TO_4_-tetrahedra, creating the ordering of the microporous framework, as well as aluminosilicate zeolite, with a difference in the T-atoms (T = Si, Al atoms for the zeolites, and T = Al, P, Si atoms for the SAPOs) [1]. Silicoaluminophosphates are unique materials since the majority of the structures are absent among the aluminosilicate zeolites, although some structures are the same as the aluminosilicate ones; for example, the CHA structure is presented both in AlSi (Chabazite zeolite) and SAPO (SAPO-34) compositions [2]. The framework topology of SAPO-34 is comprised of cylinder-like cages (6.7 × 6.7 × 10.0 Å) with 8-ring pore mouths (3.8 × 3.8 Å), and the small pore mouths only allow linear olefins and small molecules to diffuse through these. SAPO-34 is widely used in catalysis, especially in the catalytic reduction of NOx [3], methanol-to-olefins (MTO) conversion [4,5], and CO_2_ hydrogenation for light olefin production [6,7], as well as in adsorption applications, such as sorbent in adsorption heat pumps [8,9]. The last application is an alternative to conventional electrically driven systems, such as air conditioning, solar air conditioning, waste heat recovery, and domestic heat pumping. However, micrometer-sized crystals have some limitations, e.g., reduced accessibility to acid sites and diffusion restriction because of the long microporous pathways, often resulting in a fast deactivation of zeolite-containing catalysts. The use of nanozeolites can overcome this limitation. Nanozeolites have properties such as enhanced surface areas, high external-to-internal atom number ratios, short diffusion path lengths, accessible active sites, and controllable surface properties, resulting in high activities, better performances, and longer lifetimes [10].

There are several approaches to forming the nanocrystals of zeolites and zeolite-like materials through the control of the crystallization conditions and reaction mixture compositions. Among them are using an extra organic template, controlling the crystallization temperature and duration, and using ultrasonic irradiation, confined-space synthesis in the inert porous matrix, and alternative synthesis approaches, as well as post-synthesis treatment of the primary micrometer-sized zeolites, followed by recrystallization [10,11,12]. 

Among the alternative synthesis ways, the dry gel method is particularly worth highlighting [13]. The dry gel crystallization (DGC) approach is divided into two techniques, namely, steam-assisted crystallization (SAC) and vapor-phase transport (VPT). 

In the SAC technique, the initial reaction gel containing the organic template is heated to obtain a dried “gel”. Then, the dried gel is placed in autoclaves in a special vessel with a small amount of water at the bottom of the autoclave located separately from the dried gel. In the VPT technique, the organic template is mixed with water and put at the bottom of the autoclave; the dry gel, in turn, does not contain the structure-directing agent. It is believed that the lack of a liquid phase does limit mass transport and, therefore, may result in a lower rate of crystal growth. One of the benefits of using DGC in nanocrystals formation is the no need to separate the nanosized crystals from the mother liquor.

SAPO-34 can also be synthesized by dry gel conversion, especially by SAC. There is a number of papers dedicated to this SAPO-34 synthesis strategy; however, there are only a few revealing SAPO-34 nanocrystal-obtaining. Zhang et al. [14] used diethylamine (DEA) as a structure-directing agent (SDA) in the amount of DEA/Al_2_O_3_ = 2/1 synthesized SAPO-34 crystals of up to 20 µm in size at 200 °C for 5 days, both with the SAC and VPT methods. Rimaz et al. [15] also used DEA with a molar ratio of DEA/Al_2_O_3_ = 6/1, which probably made it possible to reduce the crystallization duration of the steam-assisted conversion to 6–1.5 h. Morpholine (MOR) with MOR/Al_2_O_3_ = 4/1 was used by Askari et al. [16] and Rezaei et al. [17]. In [16], the crystallization duration was reduced for 3–6 h at 200 °C, and SAPO-34 crystals of ca. 0.3–3 µm were obtained. In turn, Rezaei et al. [17] tested an enhanced crystallization temperature of 300–400 °C to form SAPO-34 crystals for 30–45 min, and the SAPO-34 crystals obtained were ca. 0.5–3 µm in size. Zhang et al. [18] also used MOR, but in a VPT conversion, and the synthesis proceeded for 5 days, resulting in large SAPO-34 crystals.

A smaller amount of SDA was used in the SAC by Di et al. [19]. They applied triethylamine (TEA) with a ratio of TEA/Al_2_O_3_ = 2.5/1 to crystallize at 170 °C for 48 h SAPO-34 plate-like nanocrystals using 30 wt.% SAPO-34 seeds, while 5–10 µm cubic crystals were formed without seeds. Finally, tetraethylammonium hydroxide (TEAOH) was used as the SDA in the steam-assisted conversion to form SAPO-34 [20,21]. Chen et al. [20] rather used a combination of the SAC and VPT techniques with varying TEAOH/Al_2_O_3_ ratios from 0.15 to 2 in the reaction mixture and added, additionally, TEA in the bottom of the autoclave and carried out the crystallization at 170 °C for 48 h, obtaining SAPO-34 crystals in the range of sizes 50–500 nm. Hirota et al. [21] crystallized SAPO-34 nanocrystals in the range of 100–150 nm at 180 °C for 1.5–24 h with TEAOH/Al_2_O_3_ = 1.8/1 without additional chemicals or seeds; however, there is no information about the texture properties of the obtained SAPO-34.

SDA mixtures are also used in dry gel conversion to obtain SAPO-34 crystals. Thus, four different templates were used in the amount of Al_2_O_3_/(TEAOH + TEA + MOR + DEA) = 1/(0.2 + 0.4 + 0.6 + 0.8) and Al_2_O_3_/(TEAOH + TEA + MOR + DEA) = 1/(0.4 + 0.2 + 0.8 + 0.6) in [22]; these complicated compositions and crystallization conditions (180 °C, 3–8 h) led to large SAPO-34 crystals of 2–10 µm.

Thus, this paper proposes the short-time crystallization method to prepare highly crystalline nanosized particles of SAPO-34 from aluminum isopropoxide or boehmite, with a small amount of organic template TEAOH and without either other additives or SAPO-34 seeds, via the steam-assisted conversion approach. 

## 2. Experimental Section

### 2.1. Materials and SAPO-34 Synthesis Procedure

Aluminum isopropoxide (AIP, Sigma-Aldrich, St. Louis, MO, USA) or boehmite (84 wt.% Al_2_O_3_), orthophosphoric acid H_3_PO_4_ (85%), and fumed SiO_2_ (Sigma-Aldrich) were used as the Al, P, and Si sources, respectively. Tetraethylammonium hydroxide (TEAOH, 25 wt.%, Sigma-Aldrich) was the organic structure-directing agent (molecular template).

Silicoaluminophosphates, SAPO-34s, were obtained via the dry gel synthesis approach in accordance with the procedure described below.

**Figure 1:**
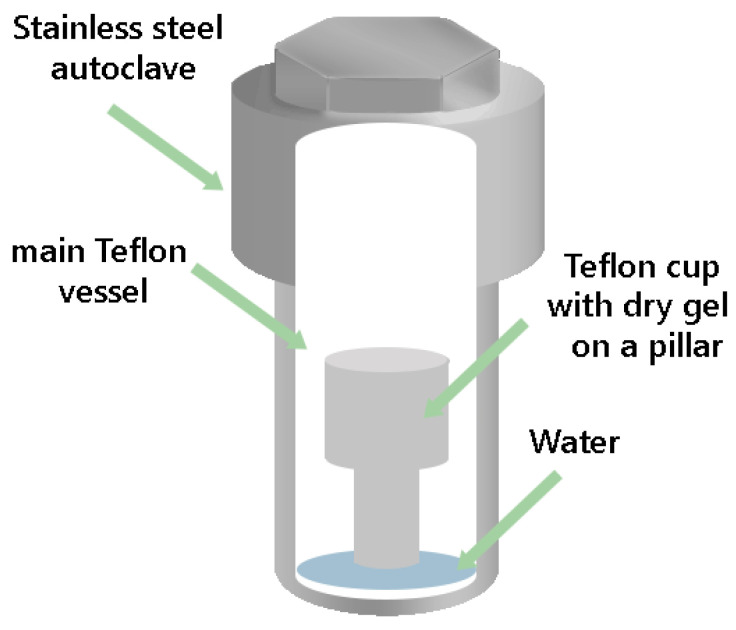
Scheme of a vessel for steam-assisted crystallization.

Initially, the gel with a molar composition of 1 Al_2_O_3_/1 P_2_O_5_/0.3 SiO_2_/1 TEAOH/50 H_2_O was prepared by the subsequent mixing of the Al source with a H_3_PO_4_ water solution; then, silica and TEAOH were added to the precursor mixture. The final gel was mixed for 3 h, followed by drying in an oven at 80 °C for 24 h to prepare the dry gel. The dry gel that was obtained was homogenized in a mortar. After, this dry gel powder was placed into a small Teflon cup on a Teflon pillar located in the main Teflon vessel in a stainless-steel autoclave. Water was added to the bottom of the main Teflon vessel, separately from the dry gel. A schematic construction of the crystallization vessel is presented in Figure 1. The water/dry gel ratio used was 5 g/3 g. Crystallization was carried out at 200 °C for 5 or 24 h. After crystallization, the product was washed with distilled water twice, dried at 100 °C, and calcined at 600 °C for 4 h with a heating rate of 3°/min.

When AIP was used, the obtained SAPOs were denoted as SAPO-34-5h and SAPO-34-24h, where “5h” and “24h” indicate crystallization time in hours. In the case of the boehmite used as the aluminum source, the sample name has an additional “B”: SAPO-34-B-5h.

### 2.2. Procedure for Obtaining SAPO-34-Covered Aluminum Heat Exchanger 

The fragment of the real aluminum plate-fin heat exchanger (HEX, Yamaha Aerox, Yamaha Corporation, Hamamatsu, Japan) was washed with a citric acid solution in an ultrasonic bath to remove Ca^2+^ and Mg^2+^ salt deposits; then, it was washed with distilled water and dried.

The SAPO-34 precursor mixture was obtained as described above (Al source = AIP) and dried to form a dry gel. To obtain the HEX-supported dry gel, a paste of precursors with a special viscosity was formed with water, adding an approximate ratio of dry gel/water = 1/3. The paste made was applied onto the lamellas of the HEX with subsequent drying at 80 °C. As a result, the HEX fragment with dry gel inside was obtained. Finally, the HEX-supported dry gel was exposed to steam-assisted crystallization at 200 °C for 5 h. After crystallization, the HEX-supported crystallization product was calcined at 600 °C to remove the TEAOH molecular template.

### 2.3. Characterization

The phase composition of the non-calcined samples was determined by X-ray diffraction (XRD) analysis with an ARLX’TRA diffractometer with CuKα radiation (wavelength was 1.5418 Å). The crystal morphology and size were studied using scanning electron microscopy (SEM) on a JSM-6460LV (JEOL) operating at an accelerated voltage of 15–20 kV. The acidic properties of SAPOs were analyzed by ammonia temperature-programmed desorption (NH_3_-TPD) on a Vra-100 mass spectrometer by an STS instrument in semi-automatic mode. The porous structure of the synthesized samples was studied by low-temperature N_2_ adsorption at 77 K on a Quantachrome Nova 1200 gas sorption analyzer. The samples were degassed at 150 °C for 3 h before the N_2_ adsorption measurement. The specific surface area, S_BET_, was calculated using the analysis of the adsorption branch of the isotherm in the relative pressure range of 0.01–0.02, based on the Brunauer–Emmett–Teller theory (BET). The total pore volume, V_tot_, was calculated from the amount of N_2_ adsorbed at a relative pressure of P/P_0_ = 0.99. The micropore volume, V_micro_, was calculated using the statistical thickness analysis of the isotherm adsorption branch and de Boer’s t-method. Mesopore distribution was performed based on the Barrett–Joyner–Halenda (BJH) equation and the desorption branch of isotherms. To evaluate the hierarchy of the SAPOs, the hierarchy factor (HF) was estimated using the formula from [23]:

HF = (S_external_/S_BET_) × (V_micro_/V_tot_), where S_external_ = S_BET_ − S_micro_

IR spectra (64 scans per spectrum, resolution 4 cm^−1^) were recorded using a Shimadzu IRAffinity-1 spectrometer at 25 °C in the 400 to 4000 cm^−1^ range. The samples were mixed and ground with KBr (SAPO-34: KBr = 3:97). Thermogravimetric analysis was performed on an automated thermogravimetric analyzer, Discovery TGA 550 (TA Instruments, Inc., New Castle, DE, USA), where the calcined sample (0.070 g) was heated from room temperature to 800 °C at a heating rate of 10 °C/min (Ar flow rate, 60 mL/min). ^27^Al and ^31^P solid-state NMR spectra were recorded using a Bruker Avance 400 pulsed Fourier spectrometer in a constant magnetic field of 9.4 T, corresponding to the resonant frequencies of 104.31 MHz (27Al) and 161.92 MHz (^31^P). The spectra were recorded at room temperature under 20 kHz magic angle spinning (MAS) conditions with the samples placed inside a standard 2.5 mm ZrO_2_ rotor. A non-selective one-pulse excitation was used in the ^27^Al NMR experiments with the π/6 pulse length of 0.5 μs and a 1 s interpulse delay. ^31^P NMR spectra were recorded using a Hahn-echo π/2-τ-π sequence with the 2.4 μs length of a π/2 pulse and a rotor-synchronized echo delay, τ, of 50 μs. A 10 s delay between the experiments was used to allow for complete magnetization relaxation. The ^27^Al spectra were referenced to a 1 M solution of Al(NO_3_)_3_ at 0 ppm, and the ^31^P NMR spectra were referenced to an 85% H_3_PO_4_ solution at 0 ppm.

## 3. Results and Discussion

### 3.1. SAPO-34 Properties

**Figure 2:**
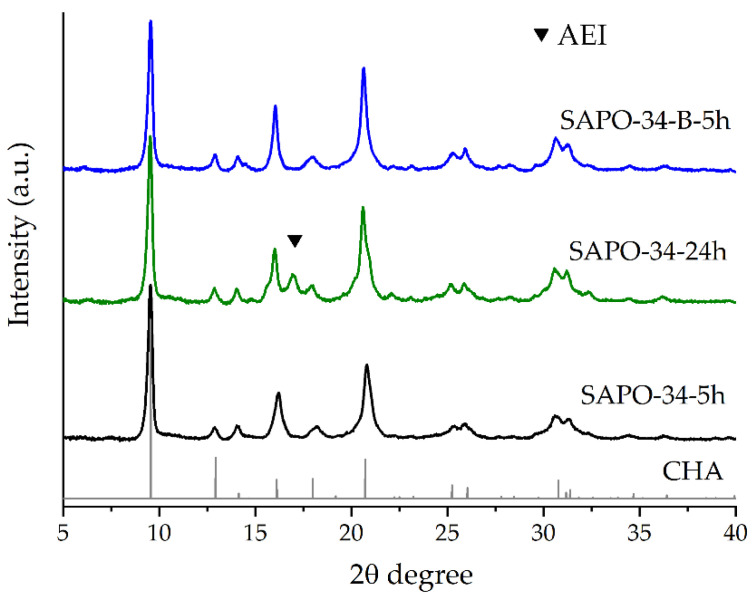
XRD patterns of synthesized SAPO-34. Triangle indicates AEI (SAPO-18) structure.

The XRD patterns (Figure 2) of the samples obtained represent a mainly CHA structure (Figure 2, the grey pattern), and some impurities of the AEI structure (SAPO-18) can be detected at ca. 17° for the SAPO-34–24h sample. It should be noted that reflections of the SAPO-34–5h sample are less intensive than in the case of the other SAPO-34 samples, and that is especially evident for the reflections at 30–32°. When the crystallization time was prolonged from 5 to 24 h, the intensity of some of the reflections was higher, and an AEI structure appeared. The AEI framework consists of layers of double 6-ring units, which are the same as those in the CHA framework. These layers are tilted in the AEI but not in the CHA structure, resulting in a pear-shaped cage with 6 8-membered rings and 12 4-membered rings [24]. The AEI structure competing with the CHA or AEI\CHA intergrowth structures may be observed [25].

Meanwhile, the width of the intensities of the SAPO-34–B–5h obtained from boehmite as the aluminum source is similar to the XRD pattern of the SAPO-34–24h obtained from the AIP. Thus, the crystallinity of the SAPO-34 from the boehmite crystallized for 5 h at 200 seems to be close to the SAPO-34 from the AIP crystallized for 24 h.

For a more detailed investigation of the SAPO-34 structure depending on the Al precursor type used, FT-IR spectroscopy, ^31^P MAS NMR and ^27^Al MAS NMR spectroscopy, and TGA were carried out for the SAPO-34–5h and SAPO-34-B-5h samples. 

**Figure 3:**
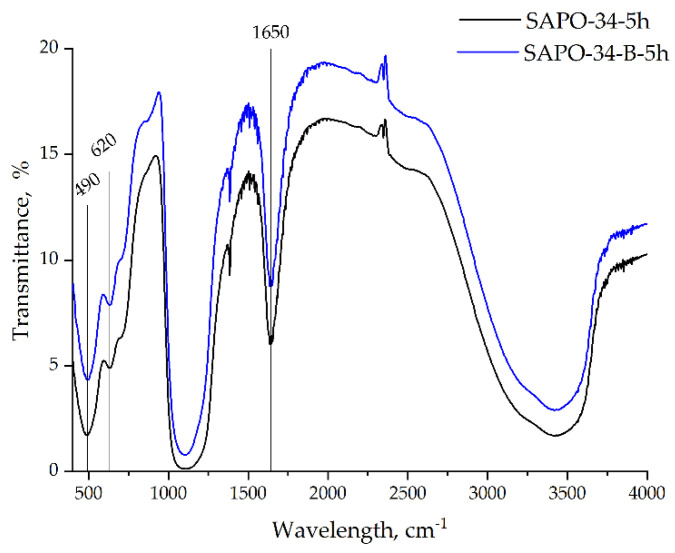
FT −IR spectra of SAPO-34 samples synthesized from AIP (SAPO-34−5h) and boehmite (SAPO-34−B−5h).

The SAPO-34–5h and SAPO-34–B–5h samples are characterized by identical sets of bands in the IR spectra (Figure 3). No bands belonging to the unreacted boehmite were detected in the IR spectrum of SAPO-34-B-5h. Vibration bands at 490 cm^−1^ and 620 cm^−1^ are ascribed to the Si–O bending of the SiO_4_ tetrahedral and the T–O (T = Si, Al, P) bending in the double-6 rings, respectively. The absorption peaks at 700 cm^−1^ and the broad-band at 1000–1250 cm^−1^ are associated with the symmetric stretching vibration of T–O and the asymmetric stretching vibration of T–O–T (Al–O–Si, Si–O–Si, and P–O–Al), respectively. The intensive band at 1650 cm^−1^ is attributed to the vibrations of physically adsorbed water molecules [26].

Finally, a broad band in the range of 3000–3700 cm^−1^ is attributed to hydroxyl groups, such as P-OH, Al-OH, Si-OH, and the bridging Si-O(OH)-Al [27].

**Figure 4:**
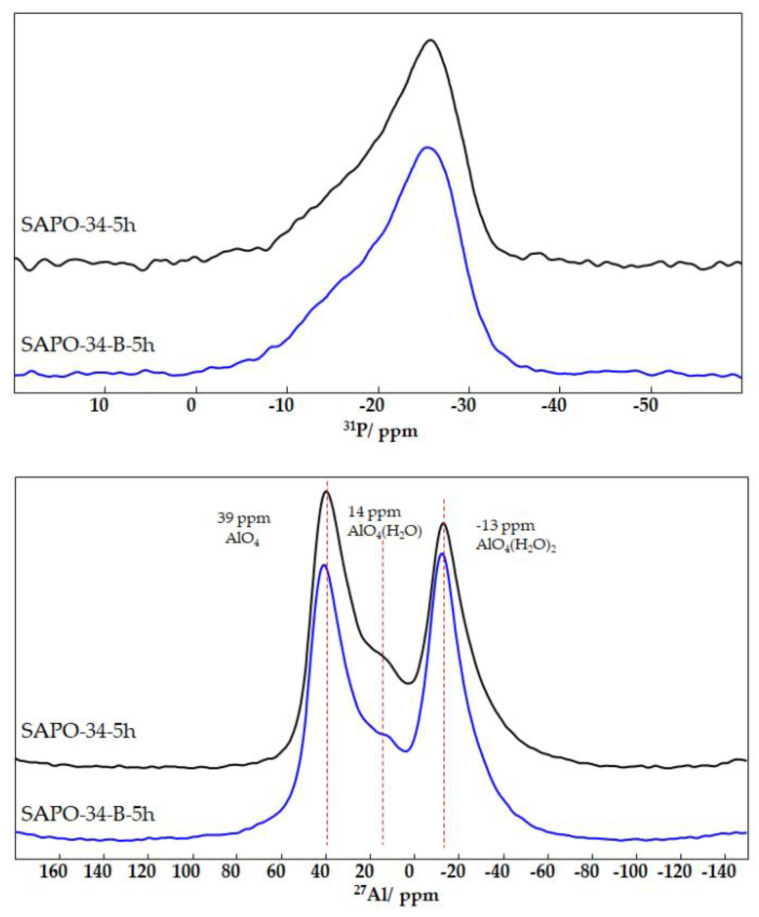
^31^P (top) and ^27^Al (down) NMR spectra of SAPO-34 samples synthesized from AIP (SAPO-34−5h) and boehmite (SAPO-34−B−5h).

The obtained ^27^Al and ^31^P MAS NMR spectra (Figure 4) fully correspond to the spectra reported for hydrated SAPO-34 [28]. The ^31^P NMR spectra of both samples are identical within the experimental error and contain a dominating line at −27 ppm that corresponds to the framework P(OAl)_4_ sites and a broad shoulder stretching from −10 to −20 ppm that corresponds to hydrated phosphorus sites. The ^27^Al NMR spectra contain three distinct lines that correspond to tetrahedrally (39 ppm), pentahedrally (14 ppm), and octahedrally (−13 ppm) coordinated Al sites. The framework alumina sites are silicoalumophosphates and possess a tetrahedral coordination, while the sites with a higher coordination arise from the presence of one or two additional water molecules in their local environment. The relative line intensities of the studied sample differ slightly with the presence of a larger amount of octahedrally coordinated sites in the SAPO-34–B–5h sample. At the same time, the amount of 5-coordinated Al sites is slightly higher in the SAPO-34–5h sample.

**Figure 5:**
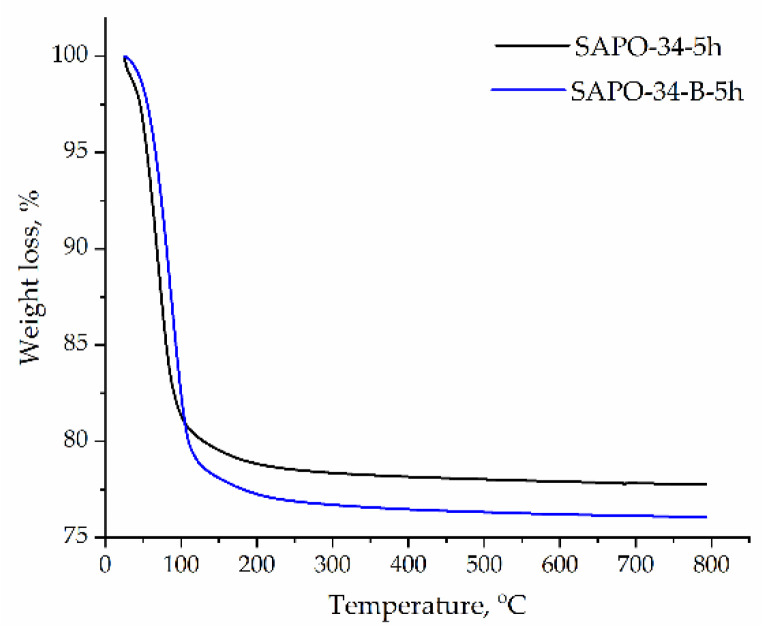
TG analysis of SAPO-34 samples synthesized from AIP (SAPO-34−5h) and boehmite (SAPO-34−B−5h).

The TGA profiles (Figure 5) of the calcined SAPO-34–5h and SAPO-34–B–5h samples show one weight loss at the temperature of up to 230 ℃ related to water desorption. The weight loss is larger in the case of the SAPO-34–B–5h sample that corresponds to the ^27^Al NMR data (more Al atoms coordinated with an additional two H_2_O molecules).

Thus, regardless of the Al precursor (AIP or boehmite), the structure of SAPO-34 is similar to each other, and only the adsorbed water amount is bigger in the case of SAPO-34–B–5h, which may be a result of a larger pore volume.

**Figure 6:**
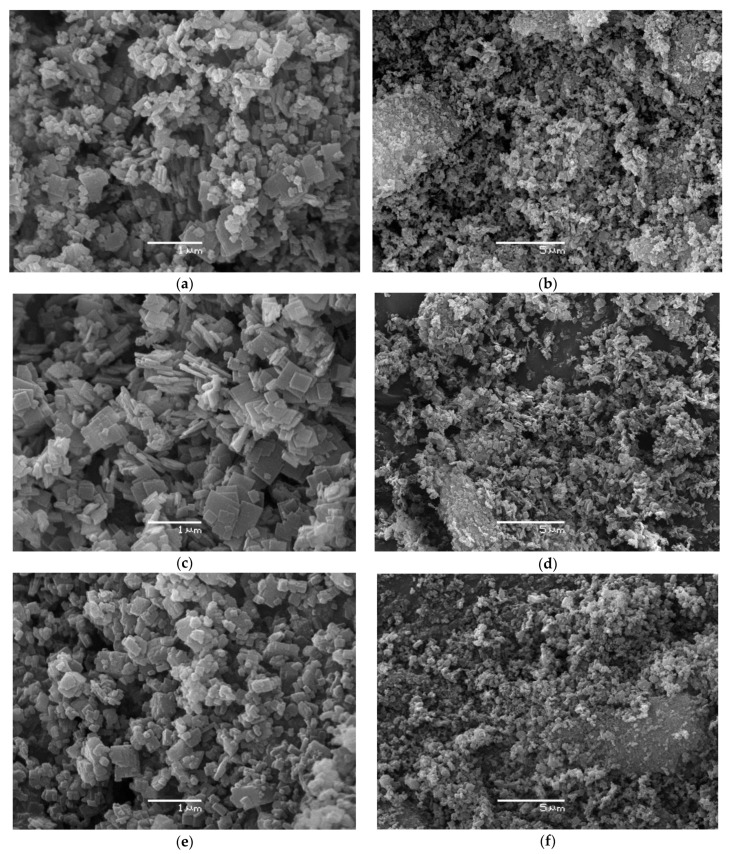
SEM images of SAPO-34 crystals: (**a**,**b**) SAPO-34–5h; (**c**,**d**) SAPO-34–24h; (**e**,**f**) SAPO-34–B–5h.

Figure 6 represents the SEM images of the obtained samples. All of the samples are nanocrystals. SAPO-34–5h (Figure 6a,b) consists of ca. 100 nm plate-like nanocrystals with sizes up to 800 nm. SAPO-34–24h (Figure 6c,d) represents mostly nano-plate crystals, while the SAPO-18 crystals are difficult to detect. The boehmite precursor led to more isometric nanocrystals of SAPO-34-B-5h. According to the images of the scanning electron microscopy, the SAPO-34 crystals, which were formed for 5 h, of the DGC are smaller in the case of using AIP than boehmite.

Thus, dry gel crystallization for 5 h at 200 °C led to SAPO-34 nanocrystals, regardless of the aluminum precursor type, in this work. Only morphology differences can be identified.

**Figure 7:**
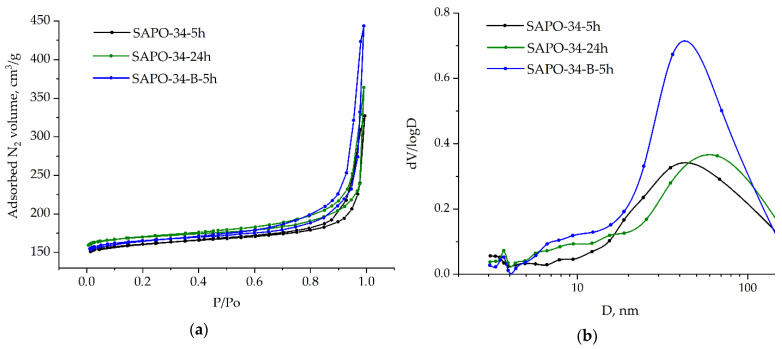
(**a**) Nitrogen sorption isotherms; (**b**) BJH pore size distribution of obtained SAPO-34.

Figure 7a represents N_2_ adsorption-desorption isotherms of the obtained samples. All of the isotherms are of the IV type, which characterizes micro-mesoporous materials. At P/P_o_ > 0.4, the capillary condensation of nitrogen arises, and the hysteresis loop of the H4 type is observed. This type of hysteresis loop describes the presence of aggregates of crystals with pores of different shapes and sizes. The larger hysteresis loop of SAPO-34–B–5h means a higher mesopore volume of the sample among all the samples in this work. Indeed, according to the BJH pore size distribution (Figure 7b), sample SAPO-34–B–5h exhibits high values of differential pore volume (dV/logD).

Additionally, the pore size distribution for the SAPO-34 samples (Figure 7b) showed distinct maxima at 3.7 nm, which is also observed in other zeolite and zeolite-like materials [29,30,31] and is attributed to the tensile strength effect of the adsorbed phase [32]. A broad distribution from 10 to 100 nm is centered at about 40 nm, representing the intercrystalline mesopores in the SAPO-34.

Table 1 shows the quantitative texture parameters of the silicoaluminophosphates. Interestingly, the micropore volume is almost the same for all of the SAPO-34 samples and amounts to 0.22–0.24 cm^3^/g; the microporosity of the SAPO-34, obtained from either the AIP or boehmite, crystallized for 5 h and is similar to each other and, apparently, slightly depends on the alumina precursor, as well as the crystallization duration, when DGC was used at the given crystallization conditions and molar composition.

Notably, the mesoporosity of the SAPO-34 samples crystallized with AIP as the aluminum source is very similar, provided that crystallization proceeded for a different time (0.29 and 0.32 cm^3^/g). Moreover, even though the crystals of the SAPO-34–5h sample are smaller than those of SAPO-34–B–5h visually (Figure 6a,e), the first is less mesoporous (0.29 vs. 0.46 cm^3^/g).

Assembling the nanocrystals is one of the ways to design hierarchical zeolites. Zeolites of this type have additional meso- and/or macro-pores. Micro- and meso-pores of uniform size are responsible for the size and shape selectivity of guest molecules and improving interactions between the host and guest molecules, whereas large meso- and macro-pores promote molecular diffusion and accessibility to the active sites. It is believed that meso-pores in a hierarchical zeolite can be present mainly in two forms: intercrystalline and intracrystalline [33]. A hierarchical zeolite with an intercrystalline mesoporous structure can be obtained by controlling the conditions to reinforce the nucleation, nanozeolite preparation, and aggregation of nanozeolite seeds with voids between them [10].

To estimate the hierarchy of the nanocrystal samples of SAPO-34, the HF was calculated for the synthesized silicoaluminophosphates. The HF is a useful tool to compare the textural properties of zeolites, showing the balance between the external surface of the sample and the volume of the micropores. It turned out that, in spite of the large mesopore volume (57–67% of the total pore volume), the HF indicates the absence of hierarchical porosity, and for all of the samples obtained in this work, the HF is in the range of 0.028–0.045 (Table 1). Thus, as shown in [23], the range of 0.05 < HF < 0.1 is typical, and HF > 0.15 is for zeolites with an intracrystalline mesoporosity formed by leaching.

Hence, the HF is less than 0.05 because the small external surface area that pointed to the nanocrystals is not highly aggregated together. However, nanocrystals are able to reduce the mass transport limitations due to the shortest diffusion path in the micropore space [11].

In general, according to texture data (Table 1), all of the samples possess a large, specific surface area in the range of 650–695 m^2^/g and are highly micro-mesoporous silicoaluminophosphates.

**Figure 8:**
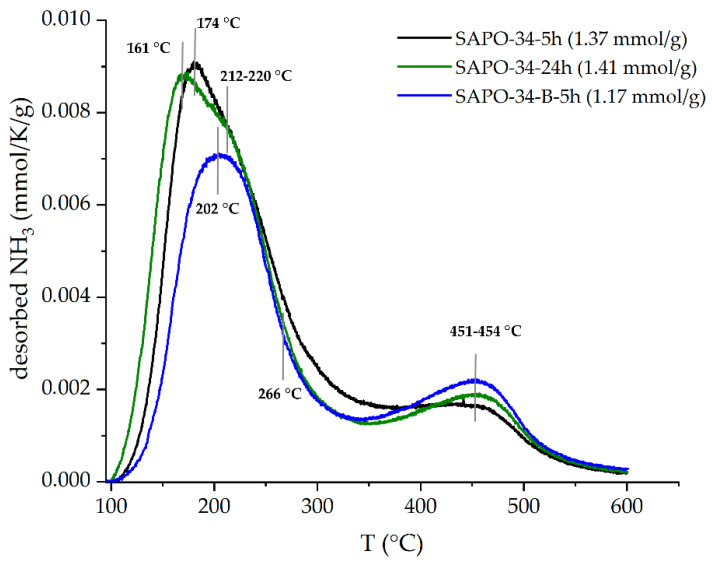
Ammonia TPD curves of SAPO-34.

The ammonia TPD curves of SAPO-34–5h and SAPO-34–24h are similar enough (Figure 8). The total amount of NH_3_ desorbed is 1.37 mmol/g and 1.41 mmol/g, respectively. At the same time, the difference in the total ammonia desorbed between the SAPO-34 crystallized for 5 h when AIP or boehmite used as the Al precursor is 14.5% (1.37 and 1.17 mmol/g, respectively). Taking into account the same silicon content in the initial gel and the dry gel synthesis approach used, which excepts the mother liquor, it can be supposed that the Si content in all of the SAPO-34s is similar. Thus, the difference in the acidity of the SAPO-34 synthesized from the AIP and boehmite may be caused by the specific Si location in the SAPO framework due to a different contribution of the SM2 and SM3 mechanisms [34].

The TPD curve of the SAPO-34 crystallized from boehmite differs from other ones of the SAPO-34 obtained from the AIP-contained precursor mixture in the temperature of NH_3_ desorption; this shift to a higher temperature indicates stronger acid sites in SAPO-34–B–5h. First, the desorption peak is at a temperature of 202 °C vs. 161–174 °C for the other SAPO-34; the second hidden peak (the shoulder) shifts from 212–220 °C to 266 °C, and the third one is detected at around 451–454 °C for all of the SAPO-34s. The low-temperature desorption peaks are attributed to the physical adsorption of NH_3_ at weak acidic sites, indicating the presence of Si–OH, P–OH, and Al–OH; the peaks at high-temperature desorption of NH_3_ indicate strong Brønsted acid sites, formed by the hydroxyl groups of Si–OH–Al.

Table 2 shows the total amount of desorbed ammonia (n, the acid site density) and individual desorption maximum.

Noteworthy, the SAPO-34 synthesized with molar TEAOH\Al_2_O_3_\SiO_2_ = 1\1\0.6 in [4] possessed two times less acidity than the SAPO-34 in this work, provided that there was two times bigger initial SiO_2_ content.

### 3.2. Primary Experiments on Obtaining Aluminum Heat Exchanger Covered by SAPO-34 

The method proposed has been tested to obtain primary experimental samples of the aluminum heat exchanger (HEX) fragment covered by SAPO-34 crystals for water sorption. 

The simplest way to apply adsorbents is by placing loose granules inside the heat exchanger (AdHEX) [35]; however, different ways to coat it are developing, such as by direct crystallization and the dip-coating approach [35,36]. Intensification of the heat flow from the adsorbent to the heat transfer liquid has been marked as a key challenge in recent years [37,38]. One of the promising ways here was to switch from a granular bed adsorber to coating the adsorbent directly onto the heat exchanger [39]. Dip-coating implies using a binder, which reduces the adsorption capacity by 10–20% due to decreasing the surface area and the pore volume. Moreover, using additional chemicals is required [40].

There are several papers devoted to the direct synthesis of SAPO-34 on supports. Bonaccorsi et al. conducted SAPO-34 synthesis on stainless-steel and aluminum supports (rectangular pieces) [41] at 200 °C for 72 h, as well as on aluminum foam [42].

**Figure 9:**
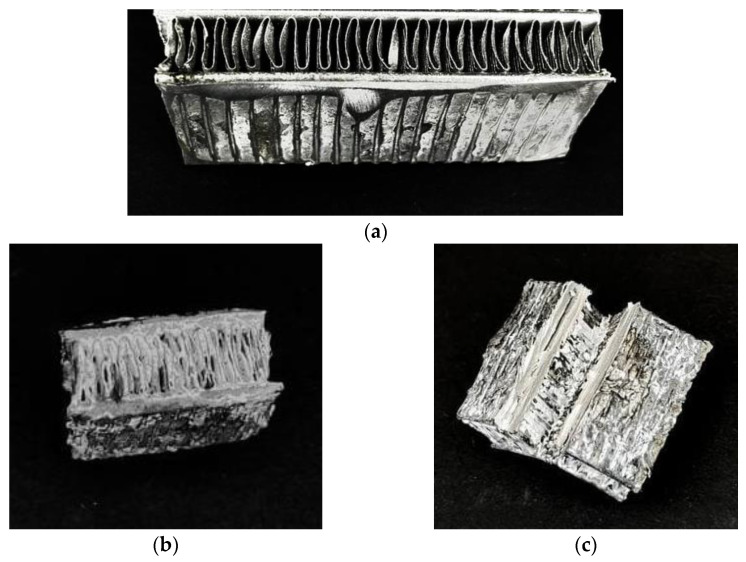
Photographs of part of (**a**) the uncovered aluminum heat exchanger; (**b**) the aluminum heat exchanger covered by SAPO-34 obtained via DGC, top view; (**c**) the aluminum heat exchanger covered by SAPO-34 obtained via SAC, dissected view.

In this work, the fragment of the initial uncovered aluminum HEX is presented in Figure 9a. After the first experiments on the in situ crystallization of the SAPO-34 via the steam-assisted approach at 200 °C for 5 h, the aluminum HEX sample was not destroyed and corroded, and white coatings of the SAPO-34 inside the HEX were observed (Figure 9b). The covering takes place through all of the lamellas, which could be seen when the HEX fragment was dissected (Figure 9c).

According to the XRD analysis (not given) of powder scraped off, the XRD pattern is similar to the SAPO-34–5h sample and presents SAPO-34.

Thus, the principal possibility of the in situ preparation of a SAPO-34-covered AdHEX via the rapid SAC method has been shown. Further, this method to obtain a covered adsorber heat exchanger will be developed to obtain uniform coverage with a certain thickness and improved mechanical properties with subsequent water vapor adsorption measurements, compared with the granulated AdHEX.

## 4. Conclusions

SAPO-34 nanocrystals with sizes of 50–150 nm were obtained via the dry gel conversion approach, namely by steam-assisted crystallization for a very short time (5 h) at 200 °C. The crystals obtained are highly crystalline with large micro-pore volumes and specific surface areas (0.22–0.24 cm^3^/g and 651–695 m^2^/g, respectively). Meanwhile, a simple reaction mixture composition with a small amount of an organic template was used. We showed that crystallization prolongation from 5 to 24 h causes an AEI (SAPO-18) structure appearance, and the micro-pore and meso-pore volumes change insignificantly. Using boehmite as the aluminum precursor led to a higher mesoporosity of the material but a little bit lower acidity compared with the samples prepared from aluminum isopropoxide. The primary experiments on obtaining the adsorber heat exchanger via the rapid method proposed were made, and this approach to obtaining heat exchangers coated with SAPO-34 is going to be developed.

Thus, the synthesis method proposed is affordable and effective for preparing highly crystalline nanoparticles of SAPO-34 with no need for post-synthetic procedures as the mother liquor separation from nanocrystals. The method also can be used to make coated adsorber heat exchangers.

## Figures and Tables

**Table 1:** Texture properties of SAPO-34.

Sample	S_BET_, m^2^/g	S_external_, m^2^/g	V_micro_, cm^3^/g	V_meso_, cm^3^/g	HF
SAPO-34–5h	651	68	0.22	0.29	0.045
SAPO-34–24h	695	52	0.24	0.32	0.032
SAPO-34–B–5h	668	57	0.23	0.46	0.028

**Table 2:** Acid properties of SAPO-34.

Sample	n NH_3_ (161–202 °C), mmol/g	n NH_3_ (212–266 °C), mmol/g	n NH_3_ (451–454 °C), mmol/g	Total Amount, mmol/g
SAPO-34–5h	0.29	0.64	0.44	1.37
SAPO-34–24h	0.27	0.77	0.37	1.41
SAPO-34–B–5h	0.60	0.24	0.33	1.17

## Data Availability

Data are available on request from the corresponding author.

## Abbreviations

AdHEX	Adsorber heat exchanger
AEI	Aluminum phosphate—Eighteen (International Zeolite Association code)
AIP	Aluminum isopropoxide
BET	Brunauer–Emmett–Teller theory
BJH	Barrett–Joyner–Halenda
CHA	Chabazite
DEA	Diethylamine
DGC	Dry gel conversion
HEX	Heat exchanger
HF	Hierarchy factor
MOR	Morpholine
MTO	Methanol-to-olefins
NH_3_-TPD	ammonia temperature-programmed desorption
SAC	Steam-assisted crystallization
SAPO	Silicoaluminophosphate(s)
SDA	Structure-directing agent
SEM	Scanning electron microscopy
TEA	Triethylamine
TEAOH	Tetraethylammonium hydroxide
VPT	Vapor-phase transport
XRD	X-ray diffraction
